# Smoking and Multidimensional Well-Being: Gender and Cohort Differences in China

**DOI:** 10.3390/bs16050755

**Published:** 2026-05-12

**Authors:** Aqsa Nadeem, Li Zheng, Xiaohe Xu, Jingyi Zhu

**Affiliations:** 1 Department of Sociology and Psychology, School of Public Administration, Sichuan University, Chengdu 610065, China; aqsanadeem@stu.scu.edu.cn (A.N.); 2023225010014@stu.scu.edu.cn (J.Z.); 2 Department of Sociology and Demography, University of Texas at San Antonio, San Antonio, TX 78249, USA; xiaohe.xu@utsa.edu

**Keywords:** smoking, well-being, gender differences, cohort effects, China

## Abstract

Smoking behavior (SB) remains a critical public health challenge in China, yet evidence on its longitudinal associations with multidimensional well-being remains limited. This study examines the relationship between smoking and three dimensions of well-being—depression, self-rated health, and happiness—using three recent waves of the China Family Panel Studies (CFPS; 2018, 2020, 2022) with 61,127 observations from 32,329 individuals. Growth curve modeling was employed to assess whether smoking is associated with well-being and whether these associations vary by gender and birth cohort. Three main findings emerge. First, smoking is positively associated with depressive symptoms (β = 0.210, *p* < 0.001) and self-rated health (β = 0.085, *p* < 0.001), but shows no significant association with happiness. Second, gender significantly moderates the relationship between smoking and well-being. The association between smoking and depressive symptoms is stronger among women, while gender differences are less pronounced for happiness. Third, cohort differences reveal substantial variation in the smoking-well-being relationship. For depression, smoking is associated with lower levels of depressive symptoms among older cohorts, but this association reverses among younger cohorts, as indicated by a significant smoking × cohort interaction (β = 0.230, *p* < 0.001). For self-rated health, the positive association between smoking and perceived health weakens across successive cohorts (β = −0.029, *p* < 0.001). No significant cohort variation is observed for happiness. These findings highlight that the relationship between smoking and well-being is shaped by gender norms and generational change. The results underscore the importance of gender-sensitive and cohort-specific tobacco control strategies, particularly in addressing the vulnerabilities of women and younger populations.

## 1. Introduction

Over the past several decades, well-being has become a central concern in both psychology and public health, encompassing psychological, emotional, and physical dimensions of human functioning (Ruggeri et al., 2020; Trudel-Fitzgerald et al., 2019; Zhang et al., 2024). This multidimensional perspective shifts attention beyond disease outcomes toward broader evaluations of life quality. Among key behavioral risk factors, smoking remains a leading cause of preventable morbidity and mortality worldwide (Chan et al., 2022), yet its implications extend beyond physical health to include mental and subjective well-being (Lappan et al., 2020).

China provides a particularly important context for examining these relationships. As the world’s largest consumer of tobacco, with over 300 million smokers, smoking represents a major and persistent public health challenge (Chan et al., 2023; N. Li et al., 2023). While prior studies have documented the adverse health consequences of smoking, much of the literature focuses on isolated outcomes, such as depression or self-rated health, rather than examining well-being as a multidimensional construct.

In addition, smoking behavior is deeply embedded in social structures, particularly gender norms and generational contexts. In China, smoking is strongly associated with masculinity and social interaction, while remaining stigmatized among women (Chen et al., 2015; Mao et al., 2015). At the same time, smoking norms have undergone substantial changes across cohorts, shaped by evolving public health policies and increasing awareness of health risks (World Health Organization, 2018; Pan et al., 2020). These shifts suggest that the relationship between smoking and well-being may not be uniform across social groups, but instead varies by gender and cohort.

Despite growing interest in smoking and well-being, several gaps remain. First, existing studies often rely on single indicators of well-being, limiting understanding of its multidimensional nature. Second, gender differences are frequently described but less often examined as moderating mechanisms in the smoking-well-being relationship. Third, although social norms surrounding smoking have changed over time, cohort variation in these associations remains underexplored. Finally, most research relies on cross-sectional data, limiting insight into how well-being varies over time.

To address these gaps, this study uses three waves of data (2018–2022) from the China Family Panel Studies (CFPS) to examine how smoking is associated with multiple dimensions of well-being over time. Focusing on depression, happiness, and self-rated health, we further investigate whether these associations differ by gender and birth cohort. By integrating a multidimensional perspective with a longitudinal design, this study provides new insights into how smoking relates to well-being across social groups and generational contexts in China.

### 1.1. Literature Review

#### 1.1.1. Association of Smoking and Well-Being

Well-being is a complex, multidimensional concept that encompasses emotional, psychological, and physical health (Sarriera & Bedin, 2017). Extensive research has consistently linked smoking to adverse outcomes across these domains, including increased risk of depression, poorer self-rated health, and reduced happiness (Park et al., 2024; Mendoza-Romero et al., 2019; Stickley et al., 2015). Numerous studies further show that smokers are more likely to experience psychological distress compared with non-smokers. For example, prior research provides robust evidence of the association between smoking and mental health risks, including anxiety and depression (Fluharty et al., 2016; Wu et al., 2023). Longitudinal findings further indicate that heavy smokers are significantly more likely to develop depressive symptoms over time (Flensborg-Madsen et al., 2011), reinforcing the consistency of this relationship across contexts.

Beyond mental health, smoking also negatively impacts both physical and subjective health outcomes. A meta-analysis by Amiri and Yaghoobi (2025) found that smokers consistently report poorer self-rated physical health compared with non-smokers. Additionally, several studies have shown that smoking detracts from overall happiness, while smoking cessation results in significant improvements in well-being (Hahad et al., 2022; Shahab & West, 2012; Taylor et al., 2014). These findings collectively affirm that smoking has a far-reaching detrimental effect on various aspects of human well-being.

#### 1.1.2. Gender, Smoking, and Well-Being

Gender differences have emerged as a significant theme in well-being research across diverse contexts. A substantial body of evidence shows that women are more likely to report higher levels of psychological distress and mental health challenges than men (Blanchflower & Bryson, 2024; Wang et al., 2017), often attributed to differences in emotion regulation, socialization, and exposure to stress (Nolen-Hoeksema et al., 2015). Beyond mental health, gender-based inequalities extend to broader dimensions of well-being, including emotional well-being and happiness (Matud et al., 2022; Short & Zacher, 2022). Taken together, these findings suggest that gender is a fundamental axis shaping variation in well-being across multiple domains, underscoring the importance of a multidimensional perspective when examining smoking-related outcomes.

Gender differences in smoking behavior are closely tied to social norms and cultural expectations. Smoking is strongly gendered across many societies, including China, where men are far more likely to smoke than women (Chen et al., 2015; T. Yang et al., 2016). Social Role Theory (Eagly & Wood, 2012) offers a useful framework for understanding these patterns, as it posits that gender roles shape not only behavioral expectations but also the social meanings attached to those behaviors. In the Chinese context, smoking is traditionally associated with masculinity, reinforcing social status and social bonding among men (Mao et al., 2015). In contrast, smoking among women is often stigmatized due to its inconsistency with prevailing norms of femininity (Liu et al., 2025). These gendered norms not only influence smoking prevalence but also shape how smoking is socially evaluated.

Beyond shaping behavior, gender also conditions how smoking is experienced and its consequences for well-being. Studies suggest that the psychosocial effects of smoking vary significantly by gender, reflecting differences in social expectations and stigma (Bottorff et al., 2014; Ozbay et al., 2020). Empirical evidence indicates that the negative impact of smoking on mental health is more pronounced among women than men, and that gendered norms may amplify the health risks and social penalties associated with smoking (Tomioka et al., 2021; L. Yang et al., 2024). Women who smoke may experience greater psychological burden due to social disapproval, whereas smoking among men may be more socially accepted or even reinforced. These findings are consistent with research highlighting gender differences in risk perception and coping strategies related to smoking (Lundborg & Andersson, 2008). Taken together, this body of work provides strong empirical support for gender differences in both smoking behavior and its differential impact on mental and physical well-being.

#### 1.1.3. Cohort Variations of Smoking and Well-Being

In addition to gender, cohort variation provides an important framework for understanding how smoking relates to well-being. As emphasized by Ryder (1965), birth cohorts capture shared historical and social experiences among individuals born in similar periods and are widely used to examine social change and generational differences. In the context of smoking, cohort differences are closely tied to shifts in public health policies, social norms, and health awareness. Across many societies, smoking has transitioned from a widely accepted social practice to an increasingly regulated and stigmatized behavior (Bayer, 2008; Stuber et al., 2008), reflecting broader changes in cultural attitudes and public health priorities (World Health Organization, 2018).

Empirical studies reveal cohort differences in the association between smoking and well-being, including depression, self-rated health, and happiness (Fergusson et al., 2003; Kubiak et al., 2019; Sargent-Cox et al., 2014). Younger cohorts, in particular, tend to report lower levels of well-being and may experience stronger psychosocial consequences associated with smoking, partly due to increasing social disapproval and stigma (Ahamed et al., 2026; Momtaz et al., 2014). These patterns are consistent with broader evidence of declining smoking prevalence and changing attitudes toward smoking in many contexts (Chen et al., 2015; World Health Organization, 2018; Pan et al., 2020). Taken together, these findings suggest that the relationship between smoking and well-being is likely to vary across cohorts, reflecting broader processes of social change in norms and health awareness.

#### 1.1.4. The Chinese Context and Research Hypothesis

Existing studies using CFPS data in China have consistently demonstrated that smoking is associated with lower subjective well-being, higher depressive symptoms, and poorer self-rated health (Chang et al., 2024; Du et al., 2022; Y. Li et al., 2022; Xu et al., 2019; Zhu et al., 2025). Furthermore, gender differences in smoking prevalence are striking, with over 50% of men and fewer than 3% of women reporting current smoking (Zhang et al., 2024). This gender disparity is consistent with cultural norms that associate smoking with masculinity (T. Yang et al., 2016). In addition, smoking behaviors and their social meanings have undergone substantial changes in China over recent decades, driven by tobacco control policies, public health campaigns, and increasing health awareness (Chen et al., 2015; World Health Organization, 2018). As a result, smoking has become progressively less socially acceptable, particularly among younger cohorts.

Building on this context, this study examines how smoking is associated with multiple dimensions of well-being across age, including depression, happiness, and self-rated health. We further investigate whether these associations vary by gender and birth cohort, thereby providing new insights into how smoking relates to well-being across social groups and generational contexts in China. We propose the following hypotheses:

**H1.**  *Smoking is associated with lower levels of well-being, including higher depression, lower happiness, and poorer self-rated health*.

**H2.**  *Gender moderates the association between smoking and well-being, such that female smokers experience less favorable outcomes than male smokers*.

**H3.**  *The association between smoking and well-being varies across birth cohorts, with stronger negative association observed among younger cohorts*.

## 2. Methods

### 2.1. Study Population and Data Source

This study utilized longitudinal data from three waves (2018, 2020, and 2022) of the China Family Panel Studies (CFPS), a nationally representative survey conducted by the Institute of Social Science Survey at Peking University. The CFPS covers a wide geographic area, including 25 provinces, municipalities, and autonomous regions, representing approximately 95% of the Chinese population. It provides comprehensive individual-level data on demographics, economic status, social behaviors, and health outcomes (Xie & Hu, 2014). The analytical sample was restricted to adults aged 18 and older who participated in at least one of the three waves. After data cleaning, the analytical sample comprised 61,127 observations from 32,329 individuals across three waves: 2018 (n = 23,522, 38.5%), 2020 (n = 19,534, 32.0%), and 2022 (n = 18,071, 29.6%). To address panel attrition, which is common in longitudinal studies, we used all available observations from the participants, allowing the data to remain unbalanced. A total of 44.9% of the observations came from individuals who participated in all three waves, 34.4% from individuals who participated in two waves, and 20.7% from individuals who participated in only one wave.

### 2.2. Measures

#### 2.2.1. Well-Being

The dependent variable in this study is well-being, conceptualized as a multidimensional construct encompassing three indicators: depression, happiness, and self-rated health (Hu et al., 2024; Yucel & Fan, 2019). Considering the measures of the three dimensions cannot be combined into one latent variable (Cronbach’s α = 0.42), they are analyzed in separate models to capture the distinct aspects of well-being rather than as a single composite score.

##### Depression

Depression was assessed using the eight-item Center for Epidemiologic Studies Depression Scale (CESD-8) (Radloff, 1977). Respondents were asked to rate the frequency of depressive symptoms over the past week on a four-point Likert scale, with higher scores indicating greater severity of depressive symptoms. The CESD-8 demonstrated acceptable internal consistency in our sample (Cronbach’s α = 0.76).

##### Happiness

Happiness was assessed via a single-item self-report question, “Do you feel happy?” with responses ranging from 0 (very unhappy) to 10 (very happy). Single-item measures of happiness are widely used in large-scale surveys and have been shown to possess acceptable validity as indicators of subjective well-being (Tadic et al., 2013; Zhou et al., 2023).

##### Self-Rated Health

Self-rated health was measured using the commonly used single-item question, “How do you think your health is?” rated on a five-point scale from 1 (very healthy) to 5 (very unhealthy). This question was reverse-coded so that higher values indicate better perceived health. It is a widely accepted proxy for overall health and has been validated in a previous study (Zou et al., 2020).

#### 2.2.2. Independent Variable

The main independent variable of the present study was smoking behavior. The CFPS survey consists of one question: “Did you smoke in the past 30 days?” A response of “Yes” was coded as 1 and “No” as 0. Those who marked “yes” were considered current smokers, and “no” indicated non-smokers in this study. This operationalization is similar to prior studies, which utilized the CFPS dataset (C. Li et al., 2021). This measure refers specifically to cigarette smoking, as defined in the CFPS survey.

#### 2.2.3. Moderating Variable

Moderators included gender (coded as 0 for female and 1 for male) and cohort (based on birth year: 0 = ≤1949, 1 = 1950–1959, 2 = 1960–1969, 3 = 1970–1979, 4 = 1980–1989, 5 = 1990–2004, treated as continuous variable). These moderators were included to examine whether the relationships between smoking and well-being differed by gender or generational cohort.

#### 2.2.4. Covariates

Control variables included age (centered at the grand mean), education (binary: 0 = high school or below, 1 = bachelor’s degree or above), log-transformed household income, marital status (0 = single, 1 = married/cohabiting), residence (0 = rural, 1 = urban), and life satisfaction (measured on a five-point scale). These variables were included as covariates to control for confounding effects and to account for other factors influencing well-being outcomes, following prior research (Hu et al., 2024; Yucel & Fan, 2019).

### 2.3. Statistical Analysis Plan

All statistical analyses were conducted using Stata 18.0. Descriptive statistics and correlation analyses were first performed to summarize the characteristics of the study variables. Given the nested structure of the data, with repeated observations (Level 1) nested within individuals (Level 2), growth curve modeling was employed (Singer & Willett, 2003). This approach appropriately accounts for within-person and between-person variation and accommodates unbalanced panel data. Age was used as the time metric and centered at the grand mean (mean age = 47.6 years). Both linear (age_c) and quadratic (age_c^2^) terms were included to capture potential non-linear age-related patterns in well-being.

A series of nested models were estimated for each outcome variable. Model 1 included age, age squared, gender, cohort, and sociodemographic covariates. Model 2 added smoking status as a time-varying predictor. Model 3 introduced an interaction between smoking and gender, and Model 4 further included an interaction between smoking and cohort to examine generational variation. All models were estimated using maximum likelihood estimation with random intercepts, allowing individuals to vary in their baseline levels of well-being while assuming a common age-related pattern across individuals.

The general form of the fitted growth curve model is expressed as:

Level 1 (Within-Person)

$${Wellbeing}_{ti}=\pi_{0i}+\pi_{1i}{smoke}_{ti}+\pi_{2i}{Age}_{ti}+\pi_{3i}{Age}_{ti}^{2}+e_{ti}$$

Level 2 (Between-person)

$$\begin{array}{l} \pi_{0i}=\beta_{00}+\beta_{01}{Female}_{i}+\beta_{02}{Cohort}_{i}+\gamma_{0i}\\ \pi_{1i}=\beta_{10}+\beta_{11}{Female}_{i}+\beta_{12}{Cohort}_{i}+\gamma_{1i}\\ \pi_{2i}=\beta_{20}+\gamma_{2i} \end{array}$$

At Level 1, well-being is modeled as a function of time-varying smoking status, age, and other covariates. Smoking is treated as a time-varying predictor, capturing within-individual changes in smoking behavior over time. At Level 2, individual differences in baseline well-being are explained by gender and cohort. A random intercept ($\gamma_{0i}$) is included to account for unobserved individual heterogeneity. Interaction terms between smoking and gender, as well as smoking and cohort, are included to assess whether the association between smoking and well-being differs across gender and birth cohorts. All slope coefficients are treated as fixed effects, indicating that the effects of smoking and age are assumed to be constant across individuals. Model fit was assessed using the Akaike Information Criterion (AIC) and Bayesian Information Criterion (BIC), with lower values indicating better fit across nested models. Statistical significance was evaluated using two-tailed tests with an alpha level of 0.05.

Multicollinearity was assessed using Variance Inflation Factors (VIF) based on a pooled OLS model. While most variables showed acceptable VIF values, age and cohort exhibited higher VIFs due to their inherent linear dependency. However, in the growth curve modeling framework, age is treated as a time-varying (within-person) variable, whereas cohort is a time-invariant (between-person) characteristic. This specification reduces concerns of multicollinearity in the estimated models.

## 3. Results

Table 1 presents the descriptive statistics for the analytical sample across the three study waves. The sample comprised 61,127 observations nested within 32,329 individuals. The mean age of participants was 47.6 years (SD = 15.9), and the sample was approximately balanced by gender (49.9% male). Current smoking prevalence was 28.4%, with a striking gender disparity: 54.2% of men reported smoking compared with only 2.6% of women (χ^2^ = 12,847.6, *p* < 0.001), consistent with Hypothesis 1. Notably, depression scores increased sharply in 2022 (Mean = 16.88, SD = 2.60) compared with 2018 (Mean = 13.53, SD = 4.00) and 2020 (Mean = 13.56, SD = 4.09), while happiness showed a slight decline over the same period (7.51 to 7.41). Self-rated health improved modestly from 2.93 in 2018 to 3.08 in 2022. Among covariates, mean life satisfaction was 4.01 (SD = 0.93), and the proportion of respondents with a bachelor’s degree or higher increased substantially across waves, from 3.1% in 2018 to 19.2% in 2022. Mean log household income increased from 9.72 to 10.02 over the study period. Approximately 80% of respondents were married or cohabiting, and 52% resided in urban areas.

Table 2 presents the Pearson correlation matrix for the key study variables: depression, happiness, and self-rated health. Significant negative correlations were observed between depression and both happiness (r = −0.30, *p* < 0.001) and self-rated health (r = −0.26, *p* < 0.001), indicating that higher depression scores are associated with lower levels of happiness and self-rated health. In contrast, a significant positive correlation was found between happiness and self-rated health (r = 0.20, *p* < 0.001), suggesting that individuals with higher self-rated health tend to report better happiness.

Table 3 presents the results of four growth curve models examining the association between smoking and depressive symptoms. Model 1 includes age, age squared, gender, cohort, and sociodemographic covariates. Age shows a significant positive association (b = 0.283, *p* < 0.001) and a negative quadratic term (b = −0.001, *p* < 0.001), indicating a nonlinear relationship between age and depressive symptoms. Male respondents report lower levels of depressive symptoms compared with females (b = −0.755, *p* < 0.001). Later birth cohorts are associated with higher levels of depression (b = 2.617, *p* < 0.001).

Model 2 adds smoking status as a time-varying predictor and shows that smoking is positively associated with depressive symptoms (b = 0.210, *p* < 0.001). Model 3 introduces the interaction between smoking and gender. The interaction term is negative and statistically significant (b = −0.539, *p* < 0.001), indicating that the association between smoking and depressive symptoms is weaker among males than females. Specifically, smoking is positively associated with depressive symptoms among females (b = 0.696, *p* < 0.001), while the association is smaller among males. Model 4 examines cohort differences by including an interaction between smoking and cohort. The interaction term is positive and significant (b = 0.230, *p* < 0.001), indicating that the association between smoking and depressive symptoms becomes stronger in more recent cohorts. For older cohorts, smoking is associated with lower levels of depressive symptoms (b = −0.408, *p* < 0.001), whereas for younger cohorts the association becomes positive.

Model fit improves across specifications, with AIC and BIC decreasing from Model 1 (AIC = 332,084.2; BIC = 332,192.4) to Model 4 (AIC = 331,978.2; BIC = 332,104.5), indicating that models including interaction terms provide a better fit to the data. The reduction in random intercept variance across models suggests that the included variables explain a meaningful portion of between-individual variation in depressive symptoms. Overall, these findings indicate that the association between smoking and depressive symptoms varies across gender and birth cohorts, with stronger associations observed among women and in more recent cohorts.

Table 4 reports the results of four growth curve models examining the association between smoking and self-rated health. Higher scores indicate better perceived health. Model 1 includes age, age squared, gender, cohort, and sociodemographic covariates. Age is negatively associated with self-rated health (b = −0.012, *p* < 0.001), with a positive quadratic term (b = 0.000, *p* < 0.001), indicating a nonlinear relationship with age. Male respondents report higher levels of self-rated health compared with females (b = 0.218, *p* < 0.001). Later birth cohorts are associated with better self-rated health (b = 0.133, *p* < 0.001).

Model 2 adds smoking status as a time-varying predictor and shows that smoking is positively associated with self-rated health (b = 0.085, *p* < 0.001). Model 3 introduces the interaction between smoking and gender. Although the interaction term is positive and significant (b = 0.170, *p* < 0.001), the main effect of smoking becomes non-significant, indicating that the association between smoking and self-rated health differs by gender. Model 4 examines cohort differences by including an interaction between smoking and cohort. The interaction term is negative and statistically significant (b = −0.029, *p* < 0.001), indicating that the positive association between smoking and self-rated health weakens across more recent cohorts. For earlier cohorts, smoking is positively associated with self-rated health (b = 0.163, *p* < 0.001), while this association becomes weaker in later cohorts.

Model fit improves across specifications, with AIC and BIC decreasing from Model 1 (AIC = 178,548.1; BIC = 178,656.4) to Model 4 (AIC = 178,497.7; BIC = 178,624.0), indicating that models including interaction terms provide a better fit to the data. The random intercept variance remains relatively stable across models, suggesting consistent between-individual variation in self-rated health. Overall, these findings indicate that the association between smoking and self-rated health varies across gender and birth cohorts, with stronger associations observed among males and weaker associations in more recent cohorts.

Table 5 reports the results of four growth curve models examining the association between smoking and happiness. Model 1 includes age, age squared, gender, cohort, and sociodemographic covariates. Age is negatively associated with happiness (b = −0.017, *p* < 0.001), with a positive quadratic term (b = 0.001, *p* < 0.001), indicating a nonlinear relationship with age. Gender is not significantly associated with happiness. Later birth cohorts report lower levels of happiness (b = −0.102, *p* < 0.001).

Model 2 adds smoking status as a time-varying predictor and shows that smoking is not significantly associated with happiness (b = −0.018, *p* = 0.840). Model 3 introduces the interaction between smoking and gender. The interaction term is not statistically significant (b = 0.006, *p* > 0.05), indicating that the association between smoking and happiness does not differ by gender. Model 4 examines cohort differences by including an interaction between smoking and cohort. The interaction term is not statistically significant (b = 0.017, *p* > 0.05), suggesting that the association between smoking and happiness does not vary across birth cohorts.

Model fit remains relatively stable across specifications, with only minor changes in AIC and BIC, indicating that the inclusion of smoking and interaction terms does not substantially improve model fit. The random effects remain unchanged across models, suggesting consistent between-individual variation in happiness. Overall, these findings indicate that smoking is not significantly associated with happiness, and this pattern is consistent across gender and birth cohorts.

**Figure 1:**
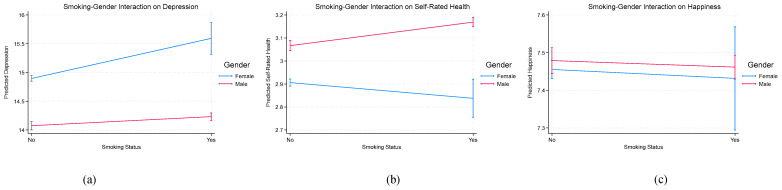
(**a**–**c**) Interaction between smoking and gender across well-being.

**Figure 2:**
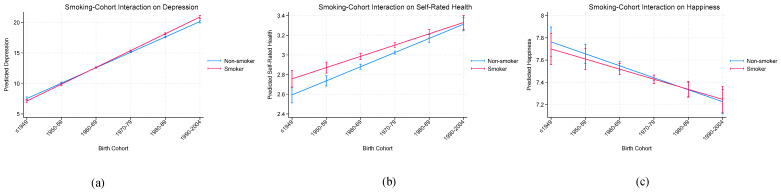
(**a**–**c**) Interaction between cohort and gender across well-being.

To facilitate interpretation of the interaction effects, predicted marginal values were calculated and plotted, as shown in Figure 1 and Figure 2. Figure 1 illustrates the moderating role of gender in the association between smoking and well-being outcomes. For depression and self-rated health, the differences between smokers and non-smokers are more pronounced among females than males, consistent with the regression results. In contrast, for happiness, the patterns remain relatively similar across gender groups, reflecting the non-significant interaction effects.

Figure 2 presents cohort differences in the association between smoking and well-being. For depression and self-rated health, the relationship between smoking and well-being varies across birth cohorts, with stronger associations observed in more recent cohorts. In contrast, no clear cohort variation is observed for happiness, consistent with the non-significant interaction effects.

## 4. Discussion

This study examined the association between smoking and multidimensional well-being, including depression, happiness, and self-rated health, using longitudinal data from the China Family Panel Studies (CFPS). The findings highlight that these associations vary across gender and birth cohorts, underscoring the importance of social context in shaping the relationship between smoking and well-being.

The results show that smoking is positively associated with depressive symptoms, which is consistent with prior research linking smoking to poorer mental health outcomes (Fluharty et al., 2016; Wu et al., 2023). At the same time, smoking is positively associated with self-rated health, indicating that smokers tend to report better perceived health despite higher levels of depression. This pattern is in line with previous studies suggesting that individuals may underestimate the long-term health risks of smoking or base their health evaluations on short-term perceptions (Krosnick et al., 2017). Notably, this positive association with self-rated health weakens across younger cohorts, reflecting shifts in public awareness and changing perceptions of smoking risks (Zhong et al., 2025).

In contrast, smoking is not significantly associated with happiness. This suggests that while smoking may be linked to mental distress and health perceptions, it does not appear to substantially influence overall subjective well-being. Given that happiness is shaped by a wide range of factors, including social relationships and life circumstances (Haller & Hadler, 2006), the absence of a significant association is consistent with prior research (Beard et al., 2014).

Gender differences play an important role in shaping the association between smoking and well-being. Consistent with Social Role Theory (Eagly & Wood, 2012), female smokers report higher levels of depressive symptoms and poorer self-rated health than male smokers. In the Chinese context, smoking among women is often stigmatized and viewed as inconsistent with prevailing gender norms (Liu et al., 2025). This mismatch may generate psychological strain through social disapproval, stigma, and reduced social support, as well as identity conflict when behavior deviates from normative expectations. These processes reflect broader gendered social expectations and power dynamics. In contrast, smoking among men is more closely aligned with socially accepted masculine norms (Kodriati et al., 2018), and may even facilitate social bonding in certain contexts. This relative social acceptance may buffer men from negative psychological consequences. Prior research similarly shows that smoking carries different social meanings across genders, with men experiencing less stigma and greater social acceptance (Stuber et al., 2008). Taken together, these findings highlight the importance of gendered social contexts in shaping the well-being consequences of smoking.

Cohort variation provides further insight into how the association between smoking and well-being has changed over time. Consistent with the cohort perspective (Ryder, 1965), the association between smoking and depressive symptoms differs across birth cohorts: it is negative among older cohorts but becomes positive among younger cohorts. This shift likely reflects changing social norms, as smoking was more socially accepted among older generations, while younger cohorts are more exposed to public health campaigns and anti-smoking norms (Pan et al., 2020). As a result, smoking may be more strongly linked to psychological strain among younger individuals. A similar pattern is observed for self-rated health. The positive association between smoking and self-rated health weakens across cohorts, suggesting that perceived health advantages have diminished over time, consistent with increased awareness of smoking risks (Adebisi et al., 2025). In contrast, no significant cohort variation is observed for happiness, indicating that its association with smoking remains relatively stable across generations.

### 4.1. Implications of the Study

The findings of this study underscore the need for gender-sensitive and cohort-specific tobacco control interventions. Given the significant well-being challenges faced by female smokers, particularly in societies where smoking is stigmatized among women, targeted interventions should focus on reducing the stigma associated with female smoking while promoting mental health support. Gender-sensitive interventions should also address the unique coping strategies and social pressures that women face, which contribute to the negative psychological consequences of smoking. For younger cohorts, interventions should focus on preventing smoking initiation by highlighting the long-term mental health risks associated with smoking, which are more pronounced in younger individuals. Public health campaigns should target the growing social stigma surrounding smoking and educate younger generations on the importance of mental health and its connection to smoking behavior. Tailored strategies, such as cessation programs aimed at younger smokers, could significantly reduce the long-term psychological burden of smoking.

### 4.2. Limitations and Future Research

Despite the strengths of this study, several limitations should be acknowledged. First, although the use of longitudinal data provides a more nuanced understanding of how smoking and well-being are associated over time, causal relationships cannot be definitively established. While longitudinal data improve temporal ordering, causal inference would be strengthened by experimental or quasi-experimental designs. Second, another limitation concerns differences in the temporal framing of key measures. Depression was assessed based on symptoms in the past week, happiness reflects current emotional state, and smoking behavior was measured over the past 30 days. These differences may capture short-term fluctuations rather than stable well-being.

Third, this study did not examine potential mediators or additional moderators of the relationship between smoking and well-being, such as social support, coping strategies, or health behaviors. Future research could explore these mechanisms to better understand the pathways linking smoking to mental and physical health outcomes. Fourth, the three well-being dimensions could not be combined into a single latent variable (Cronbach’s α = 0.42); future research should develop a validated multi-item well-being scale. Finally, although this study uses nationally representative longitudinal data from China, the findings may not be fully generalizable to other contexts. Given that smoking norms and gender roles vary across societies, future research should examine whether similar patterns emerge in different cultural and institutional settings.

## 5. Conclusions

In conclusion, this study provides new evidence on the complex relationship between smoking and multidimensional well-being, demonstrating that these associations vary systematically across gender and birth cohorts. By integrating a cohort perspective with gender-based analysis, the findings highlight that the health and psychosocial consequences of smoking are shaped by both social norms and historical context.

Rather than reflecting a uniform effect, smoking is associated with distinct patterns across different dimensions of well-being, underscoring the importance of adopting a multidimensional approach. These results contribute to a more nuanced understanding of smoking behavior in contemporary China and emphasize the need for targeted interventions that account for gender differences and generational change in smoking norms.

## Figures and Tables

**Table 1:** Sample Characteristics of Study Population by Wave (N = 61,127).

Variable	2018	2020	2022	Total Sample
Continuous Variables, M (SD)				
Age	48.22 (16.13)	46.89 (15.90)	47.70 (15.49)	47.64 (15.88)
Depression (CESD-8)	13.53 (4.00)	13.56 (4.09)	16.88 (2.60)	14.53 (3.98)
Happiness (0–10)	7.51 (2.14)	7.48 (2.08)	7.41 (2.10)	7.47 (2.11)
Self-rated health (1–5)	2.93 (1.21)	3.06 (1.20)	3.08 (1.18)	3.02 (1.20)
Life satisfaction (1–5)	4.01 (0.95)	4.02 (0.92)	4.00 (0.92)	4.01 (0.93)
Log household income	9.72 (0.99)	9.84 (0.97)	10.02 (0.98)	9.85 (0.99)
Categorical Variables, %				
Gender (male)	49.43	49.98	50.40	49.89
Current smoker	29.05	27.97	27.96	28.38
Current smoker (male)	55.54	53.59	53.26	54.24
Current smoker (female)	3.15	2.35	2.25	2.63
Education (bachelor’s or higher)	3.09	7.35	19.17	9.21
Marital Status (Married/cohabiting)	80.41	79.59	79.96	80.02
Residence (Urban)	51.87	51.32	53.28	52.11
N	23,522	19,534	18,071	61,127

Note: M = Mean. SD = Standard deviation. CESD-8 = Center for Epidemiologic Studies Depression. Happiness ranges from 0 (very unhappy) to 10 (very happy). Self-rated health ranges from 1 (very unhealthy) to 5 (very healthy). Life satisfaction ranges from 1 (very dissatisfied) to 5 (very satisfied). Education is coded as 0 = high school or below, 1 = bachelor’s degree or above. Residence is coded as 0 = rural, 1 = urban.

**Table 2:** Pearson Correlation Matrix of Key Study Variables.

Variable	1	2	3
1. Depression	1.00		
2. Happiness	−0.30 ***	1.00	
3. Self-rated health	−0.26 ***	0.20 ***	1.00

*** *p* < 0.001.

**Table 3:** Growth Curve Models for Depression (N = 61,126 Observations, 32,329 Individuals).

Variable	Model 1	Model 2	Model 3	Model 4
Fixed Effects				
Intercept	15.135 *** (0.226)	15.117 *** (0.226)	15.097 *** (0.226)	15.403 *** (0.227)
Age (centered)	0.283 *** (0.005)	0.282 *** (0.005)	0.282 *** (0.005)	0.279 *** (0.005)
Age^2^ (centered)	−0.001 *** (0.000)	−0.001 *** (0.000)	−0.001 *** (0.000)	−0.001 *** (0.000)
Gender (Male)	−0.755 *** (0.035)	−0.864 *** (0.042)	−0.822 *** (0.044)	−0.879 *** (0.042)
Cohort	2.617 *** (0.049)	2.613 *** (0.049)	2.613 *** (0.049)	2.524 *** (0.050)
Smoking (Yes)		0.210 *** (0.045)	0.696 *** (0.144)	−0.408 *** (0.080)
Smoking × Gender			−0.539 *** (0.152)	
Smoking × Cohort				0.230 *** (0.024)
Log household income	−0.285 *** (0.018)	−0.284 *** (0.018)	−0.283 *** (0.018)	−0.289 *** (0.018)
Education (High)	0.970 *** (0.058)	0.985 *** (0.058)	0.982 *** (0.058)	1.019 *** (0.058)
Marital status (Married)	−0.589 *** (0.050)	−0.589 *** (0.050)	−0.585 *** (0.050)	−0.574 *** (0.050)
Residence (Urban)	−0.564 *** (0.036)	−0.560 *** (0.036)	−0.561 *** (0.036)	−0.565 *** (0.036)
Life satisfaction	−0.937 *** (0.017)	−0.936 *** (0.017)	−0.936 *** (0.017)	−0.936 *** (0.017)
Random Effects				
var(Intercept)	4.231	4.219	4.213	4.191
var(Residual)	9.882	9.885	9.886	9.886
Model Fit				
AIC	332,084.2	332,064.5	332,053.9	331,978.2
BIC	332,192.4	332,181.8	332,180.2	332,104.5

*** *p* < 0.001. Note: Standard errors are in parentheses. Reference categories: Smoking = Non-smoker, Gender = Female, Education = Low, Marital status = Single, Residence = Rural.

**Table 4:** Growth Curve Models for Self-Rated Health (N = 61,126 Observations, 32,329 Individuals).

Variable	Model 1	Model 2	Model 3	Model 4
Fixed Effects				
Intercept	1.371 *** (0.066)	1.360 *** (0.066)	1.367 *** (0.066)	1.327 *** (0.066)
Age (centered)	−0.012 *** (0.001)	−0.012 *** (0.001)	−0.012 *** (0.001)	−0.011 *** (0.001)
Age^2^ (centered)	0.000 *** (0.000)	0.000 *** (0.000)	0.000 *** (0.000)	0.000 *** (0.000)
Gender (Male)	0.218 *** (0.011)	0.175 *** (0.013)	0.161 *** (0.014)	0.176 *** (0.013)
Cohort	0.133 *** (0.015)	0.133 *** (0.015)	0.133 *** (0.015)	0.144 *** (0.015)
Smoking (Yes)		0.085 *** (0.014)	−0.068 (0.043)	0.163 *** (0.024)
Smoking × Gender			0.170 *** (0.045)	
Smoking × Cohort				−0.029 *** (0.007)
Log household income	0.039 *** (0.005)	0.040 *** (0.005)	0.039 *** (0.005)	0.040 *** (0.005)
Education (High)	−0.076 *** (0.016)	−0.071 *** (0.016)	−0.070 *** (0.016)	−0.074 *** (0.016)
Marital status (Married)	−0.003 (0.015)	−0.003 (0.015)	−0.004 (0.015)	−0.004 (0.015)
Residence (Urban)	−0.015 (0.011)	−0.013 (0.011)	−0.013 (0.011)	−0.013 (0.011)
Life satisfaction	0.188 *** (0.005)	0.189 *** (0.005)	0.189 *** (0.005)	0.189 *** (0.005)
Random Effects				
var(Intercept)	0.589	0.589	0.588	0.589
var(Residual)	0.655	0.655	0.655	0.654
Model Fit				
AIC	178,548.1	178,511.2	178,499.0	178,497.7
BIC	178,656.4	178,628.4	178,625.2	178,624.0

*** *p* < 0.001. Note: Standard errors are in parentheses. Higher scores indicate better health. Reference categories: Smoking = Non-smoker, Gender = Female, Education = Low, Marital status = Single, Residence = Rural, Life satisfaction = Very dissatisfied.

**Table 5:** Growth Curve Models for Happiness (N = 61,126 Observations, 32,329 Individuals).

Variable	Model 1	Model 2	Model 3	Model 4
Fixed Effects				
Intercept	2.109 *** (0.111)	2.112 *** (0.111)	2.112 *** (0.111)	2.131 *** (0.112)
Age (centered)	−0.017 *** (0.002)	−0.017 *** (0.002)	−0.017 *** (0.002)	−0.017 *** (0.002)
Age^2^ (centered)	0.001 *** (0.000)	0.001 *** (0.000)	0.001 *** (0.000)	0.001 *** (0.000)
Gender (Male)	0.015 (0.017)	0.024 (0.021)	0.024 (0.021)	0.023 (0.021)
Cohort	−0.102 *** (0.024)	−0.101 *** (0.024)	−0.101 *** (0.024)	−0.108 *** (0.024)
Smoking (Yes)		−0.018 (0.022)	−0.024 (0.071)	−0.064 (0.039)
Smoking × Gender			0.006 (0.075)	
Smoking × Cohort				0.017 (0.012)
Log household income	0.130 *** (0.009)	0.129 *** (0.009)	0.129 *** (0.009)	0.129 *** (0.009)
Education (High)	0.037 (0.028)	0.035 (0.028)	0.036 (0.028)	0.038 (0.029)
Marital status (Married)	0.334 *** (0.025)	0.334 *** (0.025)	0.334 *** (0.025)	0.335 *** (0.025)
Residence (Urban)	0.065 *** (0.018)	0.065 *** (0.018)	0.065 *** (0.018)	0.065 *** (0.018)
Life satisfaction	0.960 *** (0.008)	0.960 *** (0.008)	0.960 *** (0.008)	0.960 *** (0.008)
Random Effects				
var(Intercept)	0.967	0.967	0.967	0.967
var(Residual)	2.439	2.439	2.439	2.439
Model Fit				
AIC	245,540.2	245,541.5	245,543.5	245,541.5
BIC	245,648.5	245,658.8	245,669.8	245,667.8

*** *p* < 0.001. Note: Standard errors are in parentheses. Reference categories: Smoking = Non-smoker, Gender = Female, Education = Low, Marital status = Single, Residence = Rural, Life satisfaction = Very dissatisfied.

## Data Availability

The data is openly available on the CFPS website (https://www.isss.pku.edu.cn/cfps/) accessed on 12 May 2025. Processed data for this paper can be made available upon reasonable request from the corresponding author.
